# Cascades of emotional support in friendship networks and adolescent smoking

**DOI:** 10.1371/journal.pone.0180204

**Published:** 2017-06-29

**Authors:** Cynthia M. Lakon, Cheng Wang, Carter T. Butts, Rupa Jose, John R. Hipp

**Affiliations:** 1 Department of Population Health and Disease Prevention, Program in Public Health, University of California Irvine, Irvine, California, United States of America; 2 Department of Sociology, Notre Dame University, Notre Dame, Indiana, United States of America; 3 Departments of Sociology, Statistics and EECS, University of California Irvine, Irvine, California, United States of America; 4 Department of Psychology and Social Behavior, University of California Irvine, Irvine, California, United States of America; 5 Departments of Criminology, Law and Society, and Sociology, University of California Irvine, Irvine, California, United States of America; TNO, NETHERLANDS

## Abstract

Social support from peers and parents provides a key socialization function during adolescence. We examine adolescent friendship networks using a Stochastic Actor-Based modeling approach to observe the flow of emotional support provision to peers and the effect of support from parents, while simultaneously modeling smoking behavior. We utilized one school (*n* = 976) from The National Longitudinal Study of Adolescent to Adult Health (AddHealth) Study. Our findings suggest that emotional support is transacted through an interdependent contextual system, comprised of both peer and parental effects, with the latter also having distal indirect effects from youths’ friends’ parents.

## Introduction

Studies over recent decades indicate the vital role social support plays in health [1–3]. Social support serves varied functions for health and health behavior, with emotional support generally extending to intimacy, attachment, and the ability to confide in and rely on someone, all of which contribute to feeling cared for [4]. Emotional support is one of the most relevant and yet most complex domains of social support for health, as it is related to both salutary [5] and adverse health outcomes [2,6–8].

### Peer and parental support provision

Social support from both peers and parents is a critical socialization force in adolescence. Parents are role models for adolescents as they learn social competencies, which in turn affect adolescents’ ability to form friendships [9–11]. As adolescence progresses, peer relationships gain increasing importance, becoming as salient a source of support for youth as are their parents [12]. Studies suggest that adolescents view both their friends [13–15] and parents [16–18] as primary sources of social support, including emotional support [19,20].

Peer and parental support differentially impact adolescent behavior including substance use [18,21]. Studies indicate a positive relationship between peer support and adolescent cigarette smoking [22]. Moreover, studies also find evidence of a multiplicative relationship between peer support and friends’ smoking behavior in relation to adolescent smoking, with adolescent smoking being amplified when higher levels of both peer support and peer smoking are present [22]. Emotionally supportive relationships in which friends smoke together might reinforce both a friendship and smoking behavior, as joint participation in the act of smoking may be a mechanism for adolescent social bonding. Moreover, receipt or provision of emotional support may even depend in part on adolescent friendship pairs having similar smoking behavior, as asymmetry on this dimension might be perceived as a threat to a friendship, as past studies indicate that close friends display similar smoking behavior [23–27]. As such, emotional support may have deleterious as well as positive health effects in adolescence, as it can reinforce delinquent behaviors within adolescent peer networks [24].

In contrast, studies indicate that parental support protects against both risk taking and other problem behavior in adolescence [28,29]. Parental support has been conceptualized as the perceived support and closeness received from a parent in helping an adolescent deal with a problem [18]. Studies indicate that parental support inversely relates to adolescent substance use [30], including cigarette smoking [18,22]. Parental support may be protective for adolescent problem behaviors including cigarette smoking though various mechanisms, including deterring youths’ affiliation with delinquent peers who smoke.

Premised upon these two support systems for adolescent youth—peers and parents–we focus herein on how emotional support is transacted within these relational systems of primary socialization for adolescents. We therefore consider the emotional support network among adolescents within their dominant institutional contexts, school and family, as a single structural system. Specifically, we address how the emotional support youth provide their friends diffuses through these support networks, while simultaneously accounting for youths’ perceptions of emotional support from their parents. We posit that emotional support diffuses through adolescent friendship networks through a cascade of relational interdependencies, with an adolescent’s capacity to provide emotional support to a friend being a function of his or her other relationships. The structures emerging from these interdependencies then either permit or inhibit the flow of emotional support throughout the network. We utilize a Stochastic Actor-Based (SAB) modeling approach to model this process. SAB models allow for interdependently and concurrently simulating the latent process by which adolescents decide to provide support to peers, while accounting for key network properties representing relational interdependencies, concurrent with the process by which they decide whether or not to smoke.

### Individual and structural properties of emotional support networks for support provision

Given the scant literature regarding the properties of adolescent emotional support networks, we utilize insights from studies of adolescent friendship networks to guide our examination of factors affecting youths’ decisions to support their peers. We make three key assumptions: 1) emotional support is an exhaustible and exclusive resource; 2) receipt of emotional support enables the recipient to provide more support to others; 3) some individuals differentially provide support to others, and we term them emotional *sources*, whereas others differentially receive support, and we term them emotional *sinks*. Our first assumption, that emotional support is an exhaustible resource and its provision requires an exclusive investment of time devoted to the recipient, implies a limit to how much emotional support an adolescent can provide to each friend. Youth have time and resource constraints, and support provision has its opportunity costs. As such, an adolescent can only provide a finite amount of support to a finite number of others.

If there are limits to the amount of emotional support youth can provide their friends, we would expect youth to disproportionately provide support to only certain friends. Friendship reciprocity is important for whom youth select as a friend [31] and likely is important in the decision to provide support. We posit that an adolescent is more likely to provide emotional support to a friend who has provided support in the past. One study indicated that perceived emotional support received from friends and the number of reciprocated best friendship ties in an adolescents’ network were positively related [32].

Our second assumption regarding emotional support provision is that the receipt of emotional support equips an adolescent to provide more to other youth. This may happen through modeling the behavior or is a consequence of having more emotional resources upon which to draw to then support others. The infusion of more support resources into an adolescent’s network may also result in emotional support continuing to diffuse through a network and affect the whole network, beyond just support exchanged within dyads.

Third, we assume that there may be specific types of adolescents who are salient for emotional support diffusion throughout a network, including: 1) those who are emotional support sources, in that they are able to provide emotional support to others in addition to the support they receive; 2) those who are emotional support sinks, in that although they receive much emotional support, they provide a relatively limited amount to other youth. One consequence of some youths’ seemingly limited capacity to provide support is to diminish or stop the diffusion of emotional support in a network. For such individuals, the incoming support may be spent in their own coping process and cannot be passed on. A consequence of this distinction between “sources” and “sinks” is that there may be a nonlinear relationship in which adolescents who receive small levels of emotional support are more able to provide support to others, but adolescents who receive very high levels of emotional support provide less to others.

### Consequences of emotional support flow for network structural properties

Given that research has not yet explored the dynamic properties of emotional support networks, it is unclear what the structural properties of these networks look like. If emotional support provision largely occurs within dyads, we would not observe triadic (i.e., three person) structures in a network and perhaps the flow of emotional support in a network would not be a network level process, but rather a dyadic one. Alternatively, the network may reflect the structural characteristic of local hierarchy, [see [33]], through triadic interdependency. This would imply that emotional support leaves a signature differentiating network structure; and whereas studies have found such an effect in friendship networks [31], it is an open question whether emotional support networks—which are capturing a closer bond—also behave in this fashion.

Understanding the structural properties of emotional support provision may give insight into whether diffusion of support happens hierarchically, in a downward net flow pattern, suggesting the presence of cascades of support initiated by youth at the top levels of the hierarchy of the social structure, which then move down the hierarchy. The structure of this process is important for understanding how to create social support interventions targeting adolescent smoking and leveraging dyadic or triadic structures in youths’ support networks. Capturing such a local hierarchy process in dynamic models can be measured by the combination of a transitive triplet structure [33]—think of this as a loop in which person i provides support to person j and person k, and k provides support to person j—and the *lack* of a three-cycles pattern [33]—the three-cycles pattern occurs from loops of support from person i to person j to person k back to person i.

Secondly, if emotional “sources” and “sinks” exist, then their presence should be reflected in network structural properties. For example, a structural manifestation of emotional sinks in a network might be expressed in the tendency for youth who receive relatively high amounts of emotional support to be more likely to receive additional emotional support in the future. Another structural property that we might observe is that adolescents who typically provide more support in general will be more likely to provide support to those who typically receive more support—thus, manifesting a preferential tendency within the network regarding who is providing support to whom.

A third important property to explore is an adolescent’s position in the emotional support network. We examine whether the tendency of an adolescent to form ties which increase their betweenness centrality in the support network affects emotional support provision. Betweenness centrality reflects the extent to which an adolescent falls on relational pathways linking other adolescents [33]. To the extent that support is transacted from adolescent to adolescent through the network, individuals high on betweenness centrality may occupy particularly important roles in transmitting support from one part of the network to another.

### Other determinants of emotional support provision

We also consider the effects of perceived parental support in youths’ decision to provide support to peers. Parental support plays a vital developmental role in adolescents’ capacities to provide support to their peers, as parents act as role models shaping youths’ own abilities to create friendships [11] [9]. As such, we expect adolescents who receive more emotional support from their parents to be better equipped to support their peers. Therefore, we explore the consequences of having friends who receive relatively high levels of emotional support from their own parents. We expect that youth will be more likely to provide emotional support to peers with supportive parents because youth indirectly benefit from this support through their peer’s increased capacities to provide support learned from their own parents.

The extent to which youth demonstrate depressive symptoms may also affect their ability to provide or receive support. The link between depression and social support is less clear among adolescents than it is among adults, with studies on adult samples indicating that depressive symptoms relate negatively to a cadre of social support measures [34] and negatively to the risk of major depression [35]. Other work yet finds that depression among adolescents decreases the amount of support received from peers [36]. In the current study, we examine the possibility that having depressive symptoms will affect youths’ ability to provide support to their friends, as we expect that youth exhibiting depressive symptoms will be less likely to provide support to their friends. We also examine whether having a friend who exhibits depressive symptoms relates to an adolescents’ level of support provision, as youth may be inclined to provide more support to friends exhibiting depressive symptoms.

### Factors affecting adolescents’ decisions to smoke

Concurrent with modeling youths’ decisions to provide support, we simultaneously examine their decisions to smoke, and how much they smoke. We examine the effect of peer influence, which herein is the aligning of one’s behavior such that it becomes more similar to the smoking behavior of ones’ friends. Numerous studies indicate that peer influence is a key developmental factor [37] which is often positively related to adolescent smoking [38,39]. In the current study, we conceptualize peer influence as whether adolescents are more likely to align their smoking behavior with those youth whom they perceive as providing emotional support. This is a novel conceptualization of peer influence, as our focus differs from that of prior studies which have examined the relationship between peer influence and smoking, in that we specifically consider relationships in which the person perceives the friend to be a source of emotional support. It is likely that youth who provide one another with emotional support have close friendship bonds which may act as strong conduits for peer influence.

We also consider the effects of salient domains of parental constructs for adolescent smoking, including parental support, parental monitoring, and the parental home smoking environment. Parental support has been shown to be protective for adolescent problem behavior [40] [41], including smoking [18]. Parental monitoring, another salient parental construct affecting engagement in adolescent risk behavior, encompasses numerous domains, including the supervision of children and communication between parents and youth [42,43], as well as parenting behaviors involving attending to one’s child’s activities and adjustments across situations [44]. Parental monitoring may reduce adolescent smoking behavior through the enhanced awareness parents gain from monitoring their adolescent’s behavior and the subsequent enforcement of negative sanctions. Parental monitoring has been negatively associated with adolescent smoking [45,46] and advancing in smoking stage progression [46]. Lastly, we examine the parental home smoking environment, which can be risk promotive for smoking if one or more parents smokes, and has been positively related to adolescent smoking initiation [47]. Youth exposed to smoking in the household may model parental smoking behavior and internalize parental norms condoning smoking.

The current study focuses on the diffusion of the provision of emotional support through adolescent support network ties, considering the provision of parental support concurrent with youths’ decisions to smoke. This study posits a novel conceptualization of how emotional support diffuses through adolescent friendship network structure. We are not aware of studies to date that have conceptualized the flow of emotional support through an adolescent friendship network, and in the context of key parental influences and adolescent smoking. We expect that numerous network characteristics in the emotional support provision model will be positively related to providing support, including the parental support constructs under study. Regarding the smoking behavior model, we hypothesize that the in-degree effect will be positively related to smoking. We also expect that peer influence, taking into account the emotional supportive properties of the tie, will have a positive relationship with smoking in that adolescents will tend to adopt the smoking behavior of those who provide support to them. We hypothesize that the parental constructs of support and monitoring will be negatively related to smoking, while having a parental home smoking environment that condones smoking will result in more adolescent smoking.

## Methods

### Participants

This study utilizes data from three waves of the National Study of Adolescent Health, in which students in grades 7–12 were surveyed in 1994–95 and 1995–96 [see [48]]. The Wave 1 interviews occurred in school (i.e., "In-School Survey"), the wave 2 interviews occurred at home six months later (i.e., wave 1 "In-Home Survey"), and the wave 3 interviews occurred at home one year after wave 2 (i.e., wave 2 "In-Home Survey"). Information relating to the demographic and social characteristics of the youth, their parents, and youths’ health risk behaviors including smoking was collected. Given that the saturated network sample of 16 schools for Add Health has many small schools (some of which combine middle and high school grades), we used the second largest school (*n* = 976), referred to as "Jefferson High" a rural predominantly White high school, see [49]. We did not use the largest school (*n* = 2,178), given that its size posed estimation challenges for a three-wave analysis using the SAB modeling strategy implemented in the R-based Simulation Investigation for Empirical Network Analysis (RSIENA) software package, see [50]. This study was reviewed and granted approval under exempt review by the Institutional Review Board at the University of California, Irvine. Add Health participants provided written informed consent for participation in all aspects of Add Health in accordance with the University of North Carolina School of Public Health Institutional Review Board guidelines that are based on the Code of Federal Regulations on the Protection of Human Subjects 45CFR46: http://www.hhs.gov/ohrp/humansubjects/guidance/45cfr46.html. Written informed consent was given by participants (or next of kin/caregiver) for their answers to be used in this study.

### Measures

#### Smoking behavior

At wave 1, the item measuring smoking asks: “During the past 12 months, how often did you smoke cigarettes?” (0 = never; 1 = once or twice; 2 = once a month or less; 3 = 2 or 3 days a month; 4 = once or twice a week; 5 = 3 to 5 days a week; 6 = nearly every day). At waves 2 and 3, the item asked: “During the past 30 days, on how many days did you smoke cigarettes?” measured continuously (0 = no days to 30 = 30 days). The variable we used across all three waves re-categorizes the response categories across the two items to ensure that they match the 30-day based category framing across waves: (0 = never, 1 = 1~3 days, 2 = 4~21 days, 3 = 22 or more days).

#### Emotional support

Constructing the adolescent emotional support network required a two-step procedure. First, the survey instrument asked each student to nominate up to five female and five male best friends in his or her school. Then, of each named tie, respondents were asked whether they had talked with the person about a problem during the past seven days before the survey. If the respondent answered affirmatively, this was coded as an instance of providing emotional support (and denoted as “1”). All other cases were coded as null (“0”). This information constituted the emotional support network. The outcome variable for providing emotional support is the presence or absence of providing support to each adolescent named as a friend.

#### Emotional support network effects

We control for endogenous network structural characteristics affecting emotional support provision by including several emotional support network measures. The *emotional support rate* parameters capture the expected number of change opportunities for each respondent in each period. The *out-degree* parameter measures the general tendency to provide emotional support. The *reciprocity* parameter captures the tendency for those who receive emotional support from someone to provide emotional support to that same person. *Reciprocity* signals close, symmetric friendships. The *transitive triplets* parameter captures the tendency of a respondent to provide emotional support to an adolescent who has received support from another adolescent that the respondent has provided support to in the past. Hence, if adolescent *i* provides emotional support to *k*, and *k* provides emotional support to *j*, at the next time point *i* is more likely to provide emotional support to *j* if there is a positive transitive triplets parameter. The *three-cycles* parameter can be considered a form of generalized reciprocity. Thus, if *k* provides support to *i*, and *j* provides support to *k*, then *i* is more likely to provide support to *j* in the next time point if there is a positive three-cycles parameter. Both transitive triplets and three-cycles are indicators of triadic closure [33], that is for individuals i, k, and j, if a tie exists between i and k, and i and j, there is a tie between k and j. The difference between these two indicators lies in the directionality of the provided emotional support between the three actors comprising these indicators. The *betweenness* parameter captures the general tendency in a network for adolescents to receive and provide emotional support to two adolescents who do not provide emotional support to each other. The *in-degree* centrality parameter measures the tendency to receive emotional support based on how much one is already receiving; a positive effect implies that those receiving much emotional support are more likely to receive additional emotional support. The *out-in-degree assortativity (square root)* parameter captures the tendency for those who frequently provide support to provide it to those who frequently receive support. The *in-degree activity* and *in-degree squared activity* parameters capture the tendency to provide emotional support as one receives increasing levels of emotional support.

We tested various homophily effects for predicting to whom emotional support is provided. The *same grade* parameter captures the tendency to provide emotional support to those in the same grade. The *same gender* parameter captures the tendency to provide emotional support to those of the same gender. The school is homogeneous based on race, and therefore we do not include measures of race. The *smoking similarity* parameter captures the tendency to provide emotional support to those at similar levels of smoking behavior.

**Fig 1:**
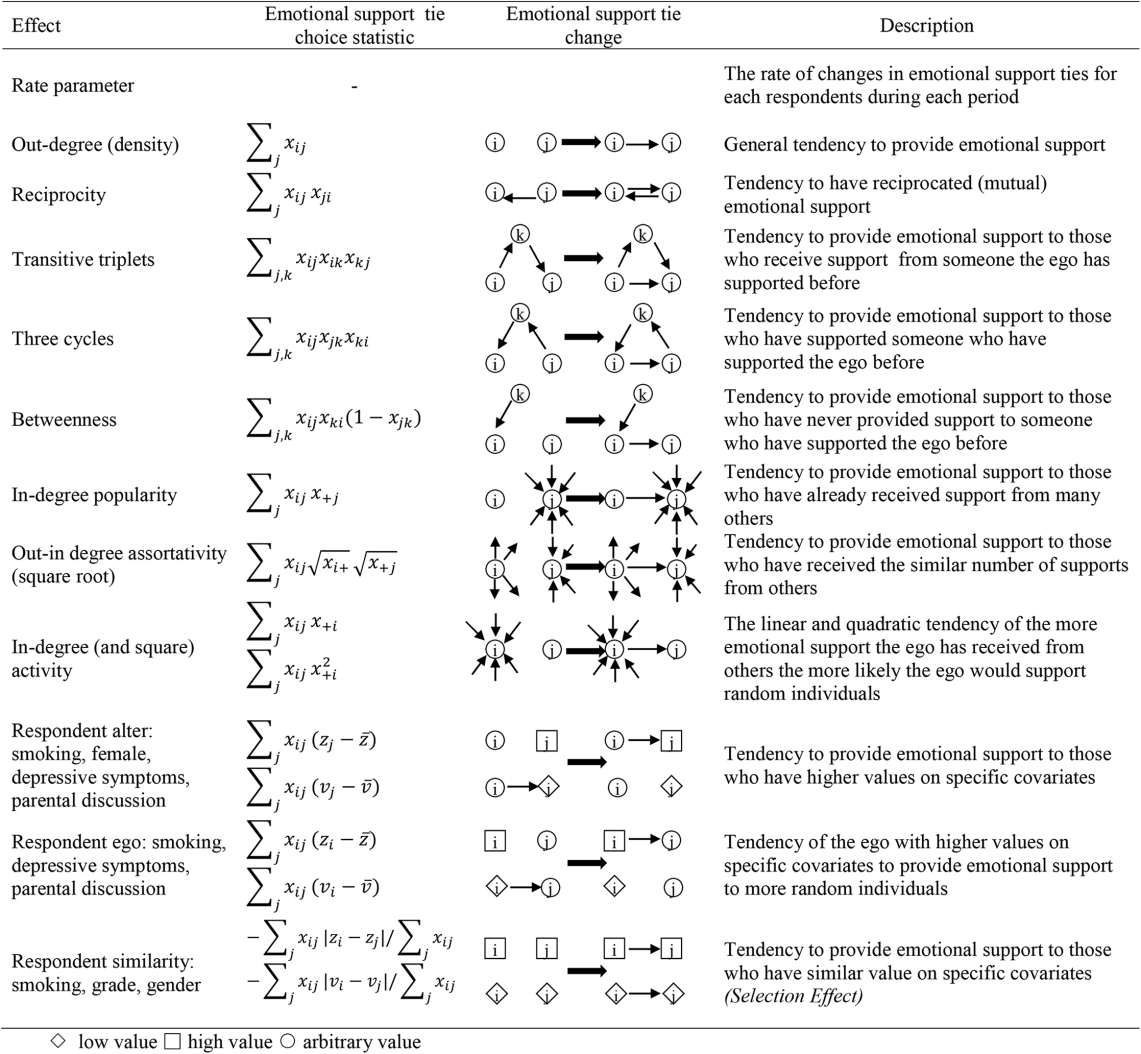
Pictorial representations of network characteristics.

**Fig 2:**
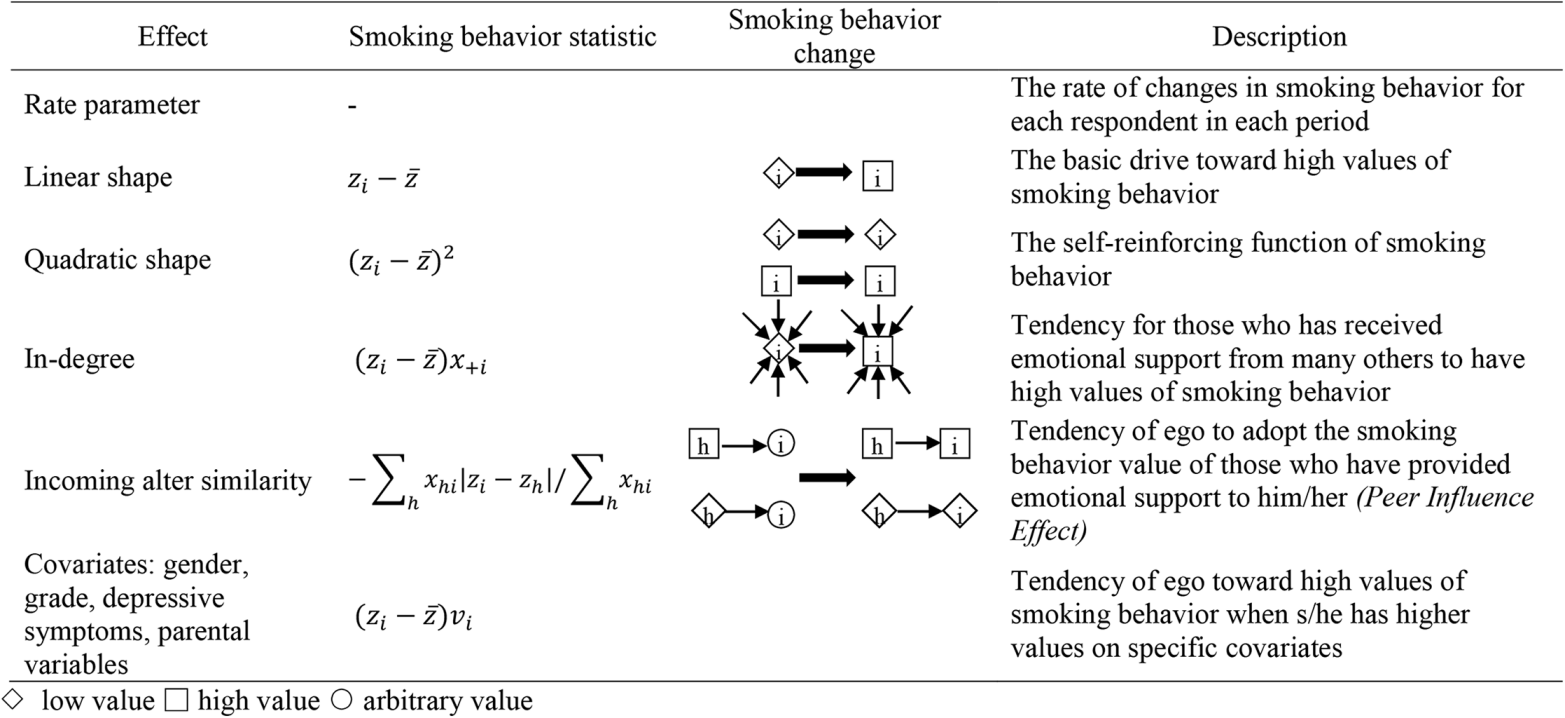
Pictorial representations of network characteristics.

We captured the tendency of certain types of adolescents to provide or receive more or less emotional support. The *smoking alter* parameter captures the tendency of those who smoke to receive emotional support. The *smoking ego* parameter captures the tendency of the respondent’s own smoking behavior to impact the amount of emotional support they provide. The *female alter* parameter captures the tendency for females to receive more emotional support than males. The *depressive symptoms ego* parameter captures the tendency of those with greater depressive symptoms to provide more emotional support, whereas the *depressive symptoms alter* parameter captures the tendency of those with greater depressive symptoms to receive more emotional support. The *parental discussion ego* parameter captures the tendency of those who discuss more issues with their parents to provide more emotional support, whereas the *parental discussion alter* parameter captures the tendency of adolescents to provide more emotional support to their friends who discuss more issues with their parents. See Figs 1 and 2 for pictorial representations of the network variables.

Due to an administrative error, some participants could only choose one male friend and one female friend during the wave 1 In-Home and wave 2 In-Home surveys. We account for this with a *limited nomination* variable (measured as: -1 = changed from full to limited nominations, 0 = no change, and +1 = changed from limited to full nominations) in the emotional support choice equations.

#### Smoking behavior effects

To capture how smoking behavior changes over time, we include several measures. The *smoking rate parameters* capture the expected number of change opportunities for smoking behavior for each respondent in each period. The *linear and quadratic shape parameters* model the effect of current smoking behavior on future smoking behavior. The smoking behavior *in-degree* parameter captures the effect of receiving more emotional support on levels of smoking behavior. The *smoking behavior incoming alter similarity* is the peer influence effect, and captures the tendency for students to change their smoking behavior such that it matches the average smoking behavior of those provide emotional support to them.

We included several measures of adolescent characteristics that might capture the tendency to change smoking behavior over time. We included a measure of *gender* (0 = male, 1 = female). We consider whether youth who exhibit depressive symptoms smoke more given that depressive symptoms positively relate to smoking [51]. We measure *depressive symptoms* as a factor score (Cronbach's α = 0.87), and is based on 19 ordinal items taken (with a few changes) from the Center for Epidemiologic Studies Depression Scale (CES-D) [52]. Age is captured as current *grade*. We used *mother’s education* as a proxy for socioeconomic status.

We measured three key domains of parental influence for adolescent smoking. *Home smoking environment* was measured by summing dichotomous measures of parent smoking behavior and cigarette availability at home. *Parental support* was estimated as a confirmatory factor analysis (CFA), with good model fit (Root Mean Squared Error of Approximation (RMSEA) = .05, and Comparative Fit Index (CFI) = .98) and we computed a standardized factor score (mean = 0, standard deviation = 1). It is based on multiple items, including whether an adolescent has talked about a personal problem with their parents (0 = no, 1 = yes), and five questions asking respondents to separately rate their mother and father to be: 1) warm and loving, 2) good communicators, 3) part of an overall "good relationship" (the response categories for all three items are: 1 = strongly disagree, 2 = disagree, 3 = neither agree nor disagree, 4 = agree, 5 = strongly agree); 4) close, and 5) caring (with the response categories for the last two items being: 1 = not at all, 2 = very little, 3 = somewhat, 4 = quite a bit, 5 = very much).

*Parental monitoring* was estimated as a CFA (the RMSEA of .06 and the CFI of .97 suggested a good fit), and we computed a standardized factor score. It combines eight questions related to the adolescent’s autonomy, with the first five related to whether the adolescent was allowed to decide: 1) their weekend curfew, 2) who they hang around with, 3) what they watched on TV, 4) how much TV they watched, and 5) their weekday bedtime (0 = no and 1 = yes, for all 7 items). The other three questions measuring parental monitoring asked whether the parent was present when the adolescent came home from school (0 = never, 1 = almost never, 2 = some of the time, 3 = most of the time, 4 = always, 5 = they brought the student home from school), went to bed (0 = never, 1 = almost never, 2 = some of the time, 3 = most of the time, 4 = always) and ate dinner (0–7 days per week).

#### Analytical strategy

In order to examine the co-evolution of emotional support provision and smoking, we utilize the Stochastic Actor-Based (SAB) model developed by Snijders and collaborators [53,54]. This routine is implemented in the RSIENA software package see [50]. In short, this model has two equations: 1) an equation in which the outcome variable is 0/1 whether the adolescent provides emotional support to each of the other adolescents in the school (analogous to a logistic regression), and 2) an equation in which the outcome is whether the adolescent increases or decreases their own smoking behavior (analogous to an ordered logistic regression). The challenge is that we do not have real time network and smoking data, but instead have three waves of longitudinal data observed at specific points in time. The Stochastic Actor-Based model is based on the assumption that network and smoking behavior data observed at each time point arise from a latent continuous time Markov process. Therefore, the model using the equations for the decision to provide emotional support to adolescents, and the decision to increase or decrease smoking behavior are simulated in continuous time based on an agent based model that is simulated with a Markov Chain Monte Carlo algorithm. The equations allow for feedback relationships between smoking and the emotional support structure. At each micro-step in the evolution of the network, an actor in the agent-based model re-evaluates his or her relationships and chooses to provide support to a new tie, continue providing support to an existing tie, or stop providing support to a tie. These choices are done based on the specified equation, and the optimal solution for determining these parameter estimates is referred to as the agent’s objective function. Concurrently, at each micro-step a behavioral decision regarding cigarette smoking is similarly modeled, with an adolescent deciding to increase, stay the same, or decrease her or his level of smoking behavior. These two equations constitute the overall objective function that is estimated. The equations also includes rate functions which capture the expected frequency of emotional support tie decisions or smoking behavior decisions made between observation points. We wrote the source code to create the smoking behavior similarity measures in C++ and compiled it in RSIENA.

## Results

### Descriptive statistics

The smoking behavior and network descriptive statistics are summarized in Table 1. At wave 1, 42% of students were non-smokers, which rose to 53% at wave 2 before falling to 45% at wave 3. At the other extreme, there were 28%, 26%, and 32% heavy smokers at the three waves, respectively. In characterizing the emotional support network, we see that about 30% of the emotional support ties were reciprocated at wave 1, and this stayed constant over the subsequent waves. The transitivity index captures the tendency for individuals who provide emotional support to the same person to provide support to one another, and was relatively stable during the study (14%~16%). The Jaccard Index measures network stability between consecutive waves. There was also a high turnover in emotional support ties, as 13% of ties persisted between the first and the second time periods in the samples, and 15% of ties persisted between the second and the third time periods. Ripley et al. (2017) point out that based on past experience with Stochastic Actor-Based modeling, there can be estimation difficulties when the Jaccard index is lower than 0.2, especially if it is less than 0.1. In this study we encountered no such estimation problems. Furthermore, the results of a post-hoc time heterogeneity test for the models found no evidence that the co-evolution of emotional support networks and smoking behavior was significantly different across the two time periods, suggesting little evidence of estimation problems. Moreover, Simpkins et al. [55] show that for larger networks a lower Jaccard index value can be tolerated. The descriptive statistics of the covariates are reported in Table 2.

**Table 1:** Behavior and network descriptive statistics.

	Jefferson High (*n* = 976)			
	Wave 1	Wave 2		Wave 3
0 = never	42.01	53.17		45.39
1 = 1-3days	21.31	9.12		11.68
2 = 4–21 days	9.02	11.58		10.55
3 = 22 or more days	27.66	26.13		32.38
Out-going ties	2,225	1,577		1,216
Reciprocity index	0.30	0.29		0.29
Transitivity index	0.14	0.16		0.16
Jaccard index	0.13		0.15	
Limited nominations (%)	0	4.82		0.41

*Note*: The reciprocity index is the proportion of ties that were reciprocal. The transitivity index is the proportion of 2-paths (ties existing between AB and BC) that were transitive (ties existing between AB, BC, and AC). The Jaccard index measures the network stability between consecutive waves.

**Table 2:** Descriptive statistics of covariates.

		Jefferson High (*n* = 976)
In-School Survey	Female (%)	48.46
	Grade level (%)	
	9th grade	28.79
	10th grade	28.48
	11th grade	21.72
	12th grade	21.00
Wave 1 In-Home Survey (Parent Survey included)	Mother education level (%)	
	Less than high school	9.18
	High school	49.62
	Some college or trade school	36.61
	Graduate of college/university	4.59
	Parent discussion (%)	58.40
	Depressive symptom, mean (SD)	0.00(0.53)
	Parental support, mean (SD)	-0.04(0.29)
	Parental monitoring, mean (SD)	-0.04(0.10)
	Home smoking environment, mean (SD)	1.42(0.73)

### Dynamic analyses

#### Emotional support choice equation

As shown in the emotional support choice equations of Table 3, similarity in smoking behavior increased the probability of providing emotional support (*b* = 0.45, *p* < .001). Thus, the odds ratio is 56.8% greater that a non-smoker will provide emotional support to another non-smoker compared to someone with a value of 1 on the smoking measure (exp(.45) = 1.568). The nonsignificant smoking ego and alter effects imply that those who smoke frequently are no more or less likely to provide or receive emotional support. There were two other homophily effects, as adolescents were 53.7% more likely to provide emotional support to others if they were the same gender (*b* = .43, p < .001) and 93.5% more likely if they were in the same grade (*b* = .66, *p* < .001).

**Table 3:** Emotional support provision and smoking models.

Effect name	beta	s.e.
Emotional support provision decision		
Friendship rate (period 1)	9.60***	0.68
Friendship rate (period 2)	10.86***	1.21
Out-degree (density)	-3.83***	0.14
Reciprocity	3.23***	0.10
Transitive triplets	1.09***	0.09
3-cycles	-0.64*	0.25
Betweenness	0.22***	0.06
In-degree—popularity	0.04*	0.01
Out-in degree^(1/2) assortativity	0.14***	0.04
In-degree–activity	0.22†	0.13
In-degree^2 –activity	-0.09***	0.02
Same grade	0.66***	0.06
Female alter	-0.04	0.05
Same gender	0.43***	0.06
Depressive symptoms alter	-0.01	0.04
Depressive symptoms ego	-0.09†	0.06
Parental discussion alter	0.23***	0.06
Parental discussion ego	0.14*	0.06
Smoking alter	0.04	0.06
Smoking ego	0.00	0.05
Smoking similarity	0.45***	0.08
Limited nomination ego	-0.12	0.12
Smoking behavior decision		
Smoking behavior rate (period 1)	8.06**	2.49
Smoking behavior rate (period 2)	12.77***	1.79
Smoking behavior linear shape	-1.67***	0.42
Smoking behavior quadratic shape	0.66***	0.03
Smoking behavior in-degree	0.04†	0.03
Smoking behavior incoming alter similarity	0.88**	0.30
Female	-0.03	0.05
Grade	-0.03	0.02
Depressive symptoms	0.13**	0.04
Mother’s education	0.01	0.02
Home smoking environment	0.15**	0.05
Parental support	-0.05	0.10
Parental monitoring	-0.24	0.22

† Two-sided p<0.1

* Two-sided p<0.05

** Two-sided p<0.01

*** Two-sided p<0.001

There were also numerous structural network effects. The negative out-degree effect implies that there is a cost to providing emotional support to others, and hence there is a strong negative effect (*b* = -3.83, *p* < .001). However, adolescents were more likely to provide emotional support to another adolescent who provided support to them as indicated by the positive reciprocity parameter (*b* = 3.23, *p* < .001). The positive transitive triplet parameter (*b* = 1.09, *p* < .001) implies that if *i* provides emotional support to *k*, and *k* provides emotional support to *j*, *i* is more likely to provide emotional support to *j* in the next time point. The negative 3-cycles parameter (*b* = -.64, *p* < .05) implies that if *j* provides support to *k*, and *k* provides support to *i*, then *i* is *less* likely to provide support to *j* in the next time point. The positive transitive triplet effect along with a negative three-cycle effect implies a tendency toward local hierarchy in the emotional support network. The positive effect for out-in degree assortativity (square root) (*b* = .14, *p* < .001) implies that adolescents with high out-degrees were more likely to provide support to those with high in-degrees, which is consistent with the notion that some adolescents are particularly needy. Likewise, the positive effect for in-degree popularity (*b* = .04, *p* < .05) implies that adolescents are more likely to provide support to those who are already receiving more support. The positive betweenness parameter (*b* = .22, *p* < .001) implies a form of “brokerage” in the emotional support network in which actors are more likely to provide emotional support to those who are not receiving emotional support from a person providing support to actors.

**Fig 3:**
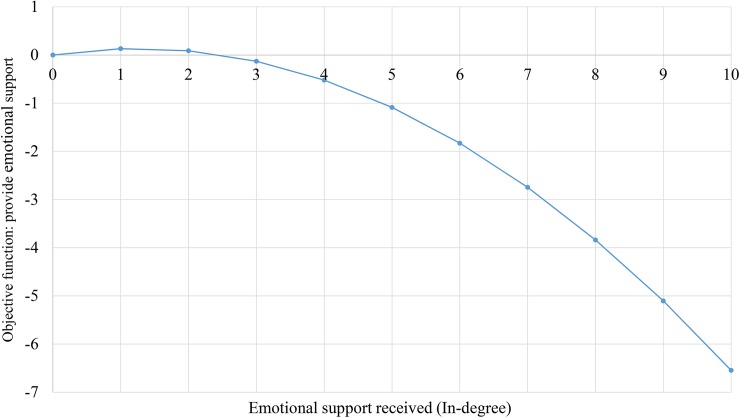
Objective function for providing emotional support, by amount of emotional support received (In-degree).

The in-degree activity and polynomial variables also demonstrate evidence of emotional support sinks. This nonlinear effect is plotted in Fig 3: the x-axis plots the in-degree capturing the number of people who are providing emotional support to the adolescent, whereas the y-axis is the objective function, which captures the probability of the adolescent providing emotional support to someone. As can be seen, for those receiving emotional support from 3 or fewer persons, there is little change in their probability of providing emotional support to someone. However, for adolescents receiving emotional support from more persons, the probability of providing emotional support drops precipitously in a nonlinear fashion. Thus, those receiving emotional support from 8 or more persons are particularly unlikely to provide emotional support to others. A reasonable proportion of the sample has these large values, as nearly 15% of the sample receives support from 5 or more, and 3.4% receive support from 8 or more.

There is evidence that those who receive more emotional support from their parents are more likely to provide emotional support to other adolescents. Thus, those who discuss more issues with their parents are 15% more likely to provide emotional support to other adolescents (*b* = .14, *p* < .05). In addition, adolescents are 26% more likely to provide emotional support to adolescents who receive more emotional support from their own parents (*b* = .23, *p* < .001). While there is no evidence that those showing more depressive symptoms receive more emotional support based on the nonsignificant depressive symptoms alter parameter, we do see modest evidence that those who show more depressive symptoms are somewhat less likely to provide emotional support (*b* = -.09, *p* < .10).

#### Smoking behavior equation

Turning to the smoking behavior equation, we find a significant positive similarity effect (peer influence effect) on smoking behavior (*b* = 0.88, *p* < .01), meaning that adolescents are more likely to adopt the smoking behavior of those who provide emotional support to them (i.e., as the smoking levels of those providing emotional support increased, so did their own smoking propensity over time). Thus, the odds are 141% greater that a non-smoker will remain a non-smoker if their support providers are non-smokers rather than smoking at a level of 1 on our smoking measure. There was modest evidence that those who receive more emotional support increase their smoking behavior over time (*b* = .04, *p* < .10). The evolution of smoking behavior was not found to vary with respondent's gender or grade. Those with more depressive symptoms smoked more over time (*b* = 0.13, *p* < .01). Although parental support and monitoring were not significantly related to smoking behavior, those with a home environment conducive to smoking increased smoking behavior over time (*b* = .15, *p* < .01).

## Discussion

Our findings are consistent with the idea that emotional support diffuses through relational interdependencies in adolescent peer networks, a system which is also affected by the broader context of not only the peer network, but also the perceived support from youths’ parents and that support which their peers perceive from their own parents. Taken together, these findings suggest that emotional support is transacted through an interdependent contextual system, comprised of both peer and parental effects, with the latter also having distal indirect effects onto youths’ friends. The provision of support to peers depended on numerous network properties, including the number of people youth provide support to, and whether or not support is reciprocated. Findings also suggest a hierarchical, unidirectional, flow of support provision, given the effects of transitive triplets and 3-cycles. The findings suggest some youth may act as sources of support, while others receive considerably more than they give, and in a non-linear fashion. We also observed homophily effects in the provision of support, with youth providing support to others in the same grade and of the same gender. Moreover, we observed evidence that parental emotional support was important: adolescents provided more support to peers if they received more emotional support from their own parent *or* to peers who received more support from their own parent. This is consistent with literature indicating that youth who have received support from their own parents are able to provide support to their friends [9,11]. We also observed a selection effect for support provision, indicating that youth had a tendency to provide emotional support to those who smoked at similar levels. Concurrently, we found that peer influence affected smoking, with youth being more likely to mimic the smoking behavior of peers who provide them emotional support. Moreover, we observed a positive relationship between a parental home environment favoring smoking and smoking behavior, mirroring past studies [31]. We also found that youth exhibiting depressive symptoms were more likely to smoke. Overall, our findings suggest a social milieu in which support provision cascades through network structures, in a hierarchical and transitive fashion, interdependently with parental support effects, which work through both direct and indirect pathways.

Given that providing emotional support entails costs likely incurred by the relational demands on the time and energy of youth, it is not surprising that we found that youth can only provide finite amounts of support to their peers and therefore tend to systematically provide emotional support to certain types of youth. For example, we observed strong homophily effects on support provision in which youth were more likely to provide emotional support to others who are similar to them along certain dimensions. Notably, adolescents were more likely to provide support to those who were more similar on smoking behavior, which is consistent with literature suggesting that adolescent peer network members have a propensity towards similarity on smoking behaviors [27,56], and perhaps this similarity is one dimension of these emotionally supportive relationships. We also observed that youth were more likely to provide support to others in their same grade or those of the same gender, which is consistent with past studies indicating both gender and age homophily among adolescent friendship pairs [56,57]. There was also evidence that adolescents were more likely to provide support to those who provide support to them (i.e., reciprocation), which is congruent with studies indicating that youth display symmetry in behavior with their friends on multiple dimensions during adolescence [56]. It is also possible that the longevity of a friendship may be contingent upon the mutual reciprocation of emotional support.

Beyond the individual level, there was evidence that key structural properties of the emotional support network offer interesting insights into how emotional support is transmitted through these networks. Our finding that transitive triplets were positively related to support provision suggests transitivity among youths’ peers in support provision, mirroring insights from Balance Theory [58]. That transitive triplets was positively related to support provision represents a more aggregated process beyond the level of the dyad, indicating a triadic structural signature of emotional support provision in these networks. To some extent, this may capture the tendency of groups to form that share in the provision of emotional support to one another. Moreover, that the three-cycles effect was also negatively related to support provision indicates that support provision may follow some hierarchical structuring in which youth at the top of the hierarchy tip off a cascade of support provision in a directional flow, likely through both reciprocated and transitive relationships. Another notable structural property was the positive betweenness effect, which is suggestive of a form of brokerage. Thus, there was a tendency for emotional support to flow from adolescent *h* to adolescent *i* to adolescent *j*, but adolescent *h* does not provide support to adolescent *j*. In this case, person *i* is in a brokerage position. This structural property is again consistent with a pattern of directional flow of emotional support in the network.

A notable pattern present throughout the analyses was that some youth were preferential receivers of support, or support sinks. Such youth received a large proportion of the emotional support resources in adolescent friendship networks, though did not provide this same amount of support to others. This was observed in numerous ways, including the positive effect of in-degree popularity—suggesting that youth were more likely to provide support to adolescents who have received support from many others—and the positive effect of out-in degree (square root) assortativity, suggesting that youth who provide relatively high amounts of support to others were more likely to provide support to those who received relatively high levels of support. This is consistent with the notion that some adolescents are particularly in need of emotional support and received support from those who are support providers. We also observed this pattern at the individual level, as we detected a nonlinear relationship in which those receiving relatively low levels of emotional support tend to provide more emotional support to others, but as the level of emotional support received increased to large amounts, the likelihood of providing emotional support decreased nonlinearly. This pattern suggests that these adolescents who receive relatively large amounts of support are differentially unlikely to provide support to others, providing further evidence of youth who act as emotional support sinks. This nonlinear decelerating effect indicates that for those receiving emotional support from three or less youth, there is little change in their probability of providing emotional support to someone else. However, for adolescents receiving emotional support from more persons, the probability of providing emotional support diminishes nonlinearly. Thus, those receiving emotional support from 8 or more persons are particularly unlikely to provide emotional support to others. This finding suggests that some youth differentially give and differentially receive large amounts of support, likely suggesting that some youth have strong capacities to give, and that other youth are in great need of emotional support.

This emotional support provided by adolescents then had important implications for adolescent smoking behavior. There was strong evidence of peer influence in which adolescents were more likely to mimic the smoking behavior of those who provided them with emotional support. This is a key finding, suggesting that an emotionally supportive tie may act as a conduit for influence to occur between adolescents and their friends. Our measurement of peer influence is likely a key innovation on the multitude of studies [59,60] which measure peer influence without taking into account whether the peers who exert this influence are also perceived as sources of emotional support. More research is necessary to better understand this measure and how it functions in relation to adolescent smoking.

Perceiving that parents are emotionally supportive, on the part of both adolescents and their friends, also had some notable effects on youths’ support provision. These findings are consistent with other research indicating that parental support affects children’s’ ability to form friendships [9,11]. Taken together, these findings indicate an indirect effect on support provision from the adolescents’ own parents to their peers. As such, adolescent support provision is likely shaped by both direct and indirect parental influences, stemming both from youths’ own families and from friends in their networks. These findings suggest that parental support works both directly and through the more distal pathway of adolescents’ peer groups.

We also found that the parental home smoking environment was relevant for increasing smoking behavior. This finding is consistent with other studies indicating that a parental home environment which condones smoking increases adolescent smoking [31] and with studies indicating that parental smoking is positively related to adolescent smoking [47,61]. Perhaps the parental home smoking context of youth exerts its influence on youth through modeling of parental smoking or adoption of parental norms, among other possible mechanisms. Lastly, youth exhibiting depressive symptoms were more likely to smoke. This finding is consistent with past studies indicating a positive relationship between depressive symptoms and adolescent smoking [31,62].

### Implications

Our findings indicate that support is transacted in networks with distinguishable network structural characteristics, and that some youth disproportionately receive or give support. Future research merits studying the diffusion of other types of support though adolescent friendship networks, perhaps companionate or instrumental support. Findings also warrant testing these study questions in a larger and more heterogeneous sample. In addition, our findings suggest the importance of considering the emotionally supportive aspects of a tie as a characteristic affecting the transmission of peer influence, and as it relates to smoking behavior. As such, future studies might consider whether peer influence is differentially transacted in emotionally supportive ties versus those that do not function to provide such support.

There are also practical implications of our findings. First, youth who are support sources may be targeted as influential for other youth, and anti-smoking norms and messages might be disseminated through these youth as such messages are likely to cascade down the social hierarchy. Our evidence that youth are likely to mimic the smoking behavior of those providing emotional support to them suggests that these youth may be important conduits of peer influence. Our findings also suggest targeting youth in transitive triadic structures for both support provision and anti-smoking messages, as these triads may be strong and viable sources of support, as the support provision is distributed across three people. These triadic structures may also act as localized areas in a network of strong peer influences, and could be viewed as key structures within which to target and initiate the diffusion of anti-smoking peer influences. Findings also suggest targeting youths’ parents and their friends’ parents with empathy training to enhance the support they provide to their adolescent youth, and simultaneously to discourage these parents from fostering a home environment which condones smoking. As such, ecological intervention approaches, simultaneously targeting youth, their parents, their home environment, and their friends’ parents, are warranted.

This study has some limitations. First, the name generator item utilized in this study was limited to naming up to 5 female and 5 male friends; our study findings would have been different if the youth in our sample could have nominated all of their friends. The self-reported smoking measures we utilized are likely subject to social desirability and recall biases. Moreover, our study does not utilize a biological measure of smoking, which would be useful in validating adolescents’ self-reports of smoking. The Add Health study follows the adolescents under study over three time points during a year and half time period; therefore we are only able to observe the co-evolution of the network and adolescent smoking over this relatively short time period. A longer observation time would provide more insight into the evolution of these processes. Also, we did not have access to measures of other types of support for both youths’ friends and parents, such as sources of instrumental, companionate, or informational support, which would each offer greater insight into the support processes transacted among these youth and the support youth perceive their parents to give. Lastly, our data are from one high school that was rural and predominantly White, so it is likely that our findings might generalize to other schools with similar characteristics. It is unclear however how our findings would generalize to similarly sized high schools in urban and suburban areas with more racially heterogeneous populations.

## Conclusion

In sum, this study investigated the coevolving processes of emotional support provision and smoking behavior among adolescent youth in one school. We found evidence that emotional support provision diffuses through key network structures, including transitive triads. We also found evidence that some youth serve disproportionately as providers and other youth as receivers of emotional support. Our findings also suggest that youths’ parents and peers play a vital role in youths’ capacities to provide support to others. Regarding smoking behavior, our findings suggest the merit of considering whether a tie is emotionally supportive and may act as a conduit for diffusing peer influence on smoking behavior. We also observed that the parental home smoking environment is a critical factor shaping smoking among youth, with parental home environments condoning smoking increasing smoking behavior among these youth. Overall, our findings suggest that emotional support provision cascades through a social system consisting of friends, parents, and youths’ friends’ parents, and one in which some youth disproportionately support other youth.
